# Crystal structure and Hirshfeld surface analyses, crystal voids, inter­molecular inter­action energies and energy frameworks of 3-benzyl-1-(3-bromoprop­yl)-5,5-di­phenyl­imidazolidine-2,4-dione

**DOI:** 10.1107/S2056989024009228

**Published:** 2024-10-04

**Authors:** Houda Lamssane, Amal Haoudi, Badr Eddine Kartah, Ahmed Mazzah, Joel T. Mague, Tuncer Hökelek, Youssef Kandri Rodi, Nada Kheira Sebbar

**Affiliations:** a Laboratory of Applied Organic Chemistry, Sidi Mohamed Ben Abdellah University, Faculty Of Science And Technology, Road Immouzer, BP 2202 Fez, Morocco; b Laboratory of Plant Chemistry Organic and Bioorganic Synthesis Faculty of Sciences Mohammed V University in Rabat 4 Avenue Ibn Battouta BP 1014 RP Morocco; c Science and Technology of Lille USR 3290, Villeneuve d’ascq cedex, France; d Department of Chemistry, Tulane University, New Orleans, LA 70118, USA; e Department of Physics, Hacettepe University, 06800 Beytepe, Ankara, Türkiye; f Laboratory of Organic and Physical Chemistry, Applied Bioorganic Chemistry Team, Faculty of Sciences, Ibnou Zohr University, Agadir, Morocco; Texas A & M University, USA

**Keywords:** crystal structure, imidazolidine, C—H⋯π(ring) inter­action, hydrogen bond, Hirshfeld surface

## Abstract

The title mol­ecule, C_25_H_23_BrN_2_O_2_, adopts a cup shaped conformation with the distinctly ruffled imidazolidine ring as the base. In the crystal, weak C—H⋯O hydrogen bonds and C—H⋯π(ring) inter­actions form helical chains of mol­ecules extending along the *b*-axis direction that are linked by additional weak C—H⋯π(ring) inter­actions across inversion centres. The Hirshfeld surface analysis of the crystal structure indicates that the most important contributions for the crystal packing are from H⋯H (51.0%), C⋯H/H⋯C (21.3%), Br⋯H/H⋯Br (12.8%) and O⋯H/H⋯O (12.4%) inter­actions. The volume of the crystal voids and the percentage of free space were calculated to be 251.24 Å^3^ and 11.71%, respectively, showing that there is no large cavity in the crystal packing. Evaluation of the electrostatic, dispersion and total energy frameworks indicate that the stabilization is dominated by the dispersion energy.

## Abstract 2

The title mol­ecule adopts a cup-shaped conformation with the distinctly ruffled imidazolidine ring as the base. In the crystal, weak C—H⋯O hydrogen bonds and C—H⋯π(ring) inter­actions form helical chains of mol­ecules extending along the *b*-axis direction, which are linked by additional weak C—H⋯π(ring) inter­actions across inversion centres.

## 1. Chemical context

Heterocyclic compounds are essential in medicinal chemistry as they serve as the basic building blocks for many biologically active mol­ecules, and thus are crucial for medication research and development (Negi *et al.*, 2020 ▸; Pradeep *et al.*, 2023 ▸). Hydantoins are a class of heterocyclic organic compounds that have piqued the inter­est of researchers due to their diverse biological applications (Aqeel *et al.*, 2023 ▸). These substances have a variety of pharmacological characteristics, such as anti­convulsant (Emami *et al.*, 2021 ▸), anti­bacterial (Pandeya *et al.*, 2000 ▸; Sangeetha *et al.*, 2016 ▸), anti­diabetic (Salem *et al.*, 2018 ▸), anti­tumor (Żesławska *et al.*, 2021 ▸), anti­nociceptive and anti-inflammatory activities (Abdel-Aziz *et al.*, 2016 ▸; Da Silva Guerra *et al.*, 2011 ▸). Phenytoin is a widely recognized pharmaceutical drug belonging to the hydantoin class and is recognized in the treatment of epileptic seizures. However, it can also be used to treat heart rhythm disorders resulting from digitalis glucoside intoxication (Dylag *et al.* 2004 ▸; Thenmozhiyal *et al.*, 2004 ▸). Continuing our research in this field, we have synthesized the compound 3-benzyl-1-(3-bromo­prop­yl)-5,5-di­phenyl­imidazolidine-2,4-dione by reacting 1,3-di­bromo­propane with 3-benzyl-5,5-di­phenyl­imidazolidine-2,4-dione under phase-transfer catalysis conditions. We determined its mol­ecular and crystal structures, performed a Hirshfeld surface analysis, and investigated its crystal voids, inter­molecular inter­action energies and energy frameworks.
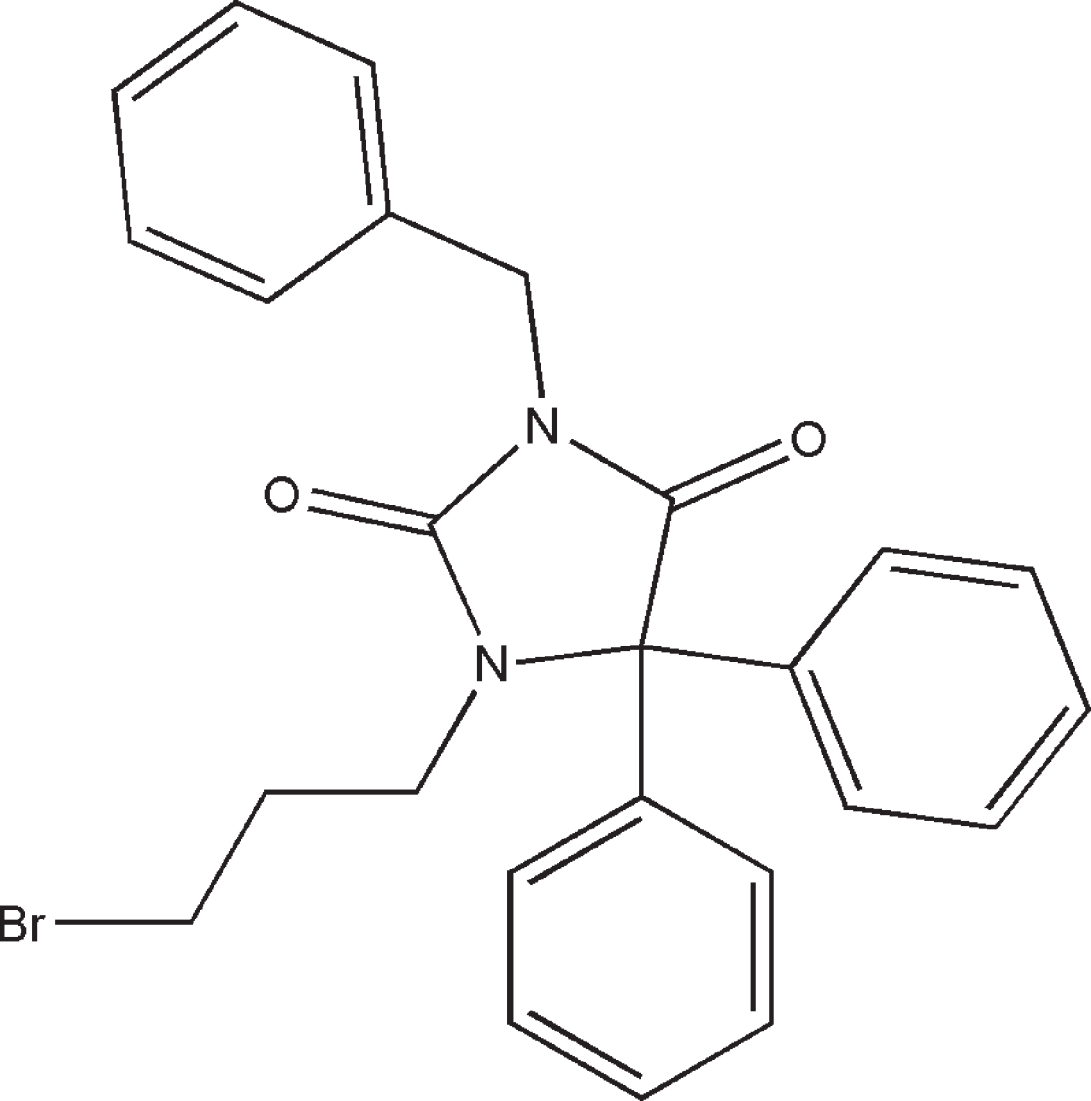


## 2. Structural commentary

**Figure 1:**
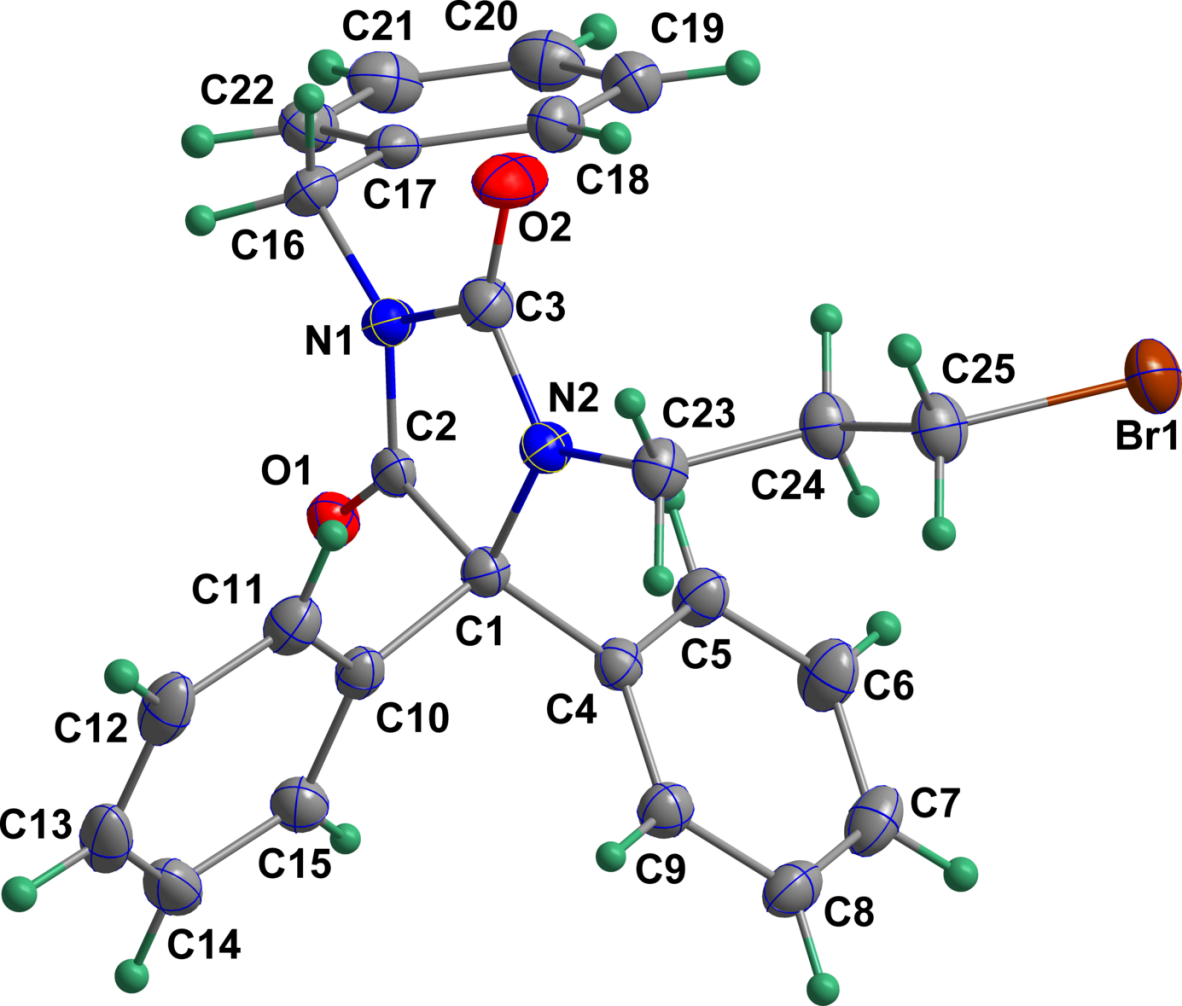
The title mol­ecule with labelling scheme and 50% probability ellipsoids.

The mol­ecule adopts a cup-shaped conformation with the imidazolidine ring as the bottom and the benzyl, the C4–C9 phenyl and the 3-bromo­propyl groups forming the sides (Fig. 1 ▸). The imidazolidine ring is quite ruffled and a puckering analysis of its conformation (Cremer & Pople, 1975 ▸) gave the parameters *Q*(2) = 0.0937 (13) Å and φ(2) = 232.0 (8)°. The best descriptor is a twist on C2—C1. The dihedral angles between the mean planes of the C4⋯C9 and C10⋯C15 rings and that of the imidazolidine ring are 78.40 (5) and 82.90 (4)°, respectively. The mean plane of the C17–C22 ring is inclined to that of the imidazolidine ring by 85.94 (4)°. All bond distances and inter­bond angles appear as expected for the formulation given.

## 3. Supra­molecular features

**Figure 2:**
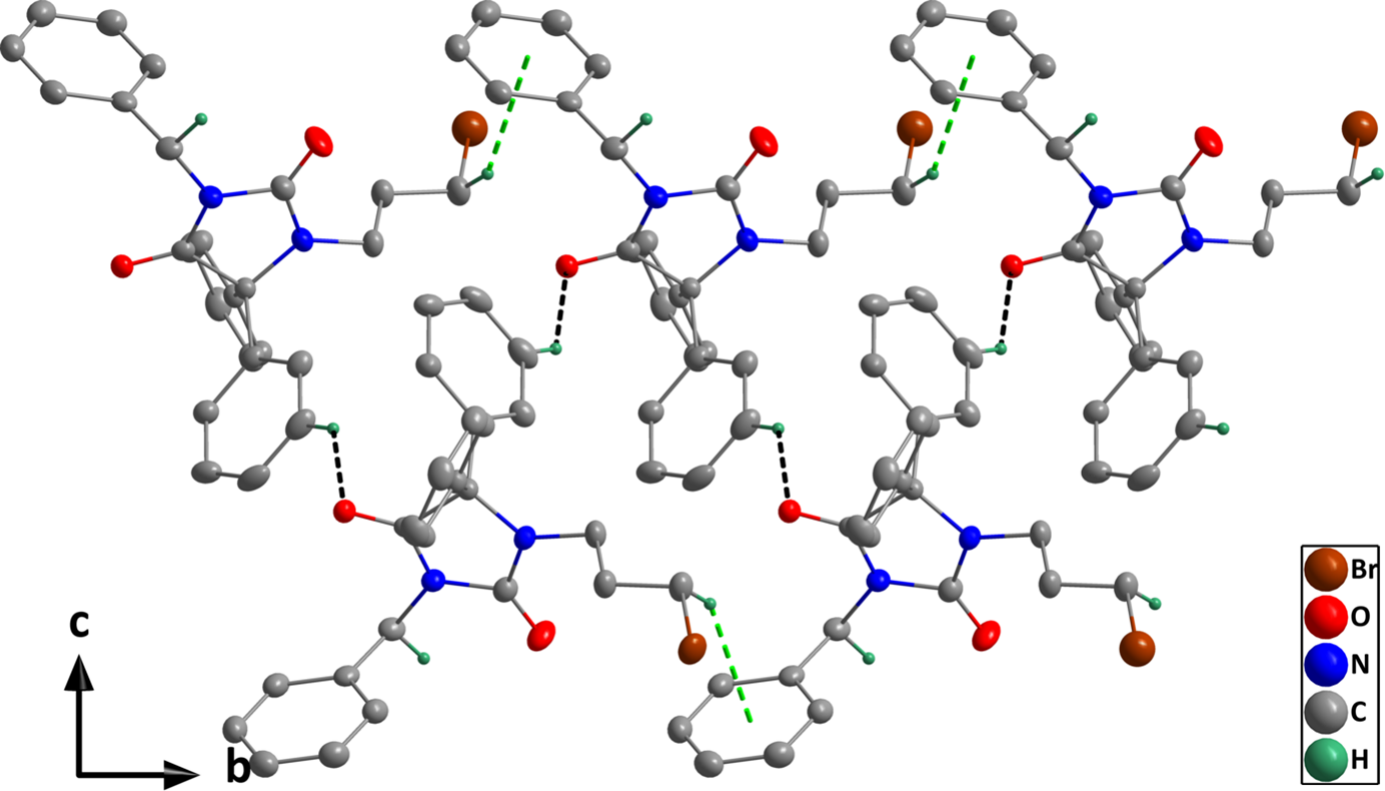
A portion of one chain viewed along the *a*-axis direction with C—H⋯O hydrogen bonds and C—H⋯π(ring) inter­actions depicted, respectively, by black and green dashed lines. Hydrogen atoms not involved in these inter­actions are omitted for clarity.

**Figure 3:**
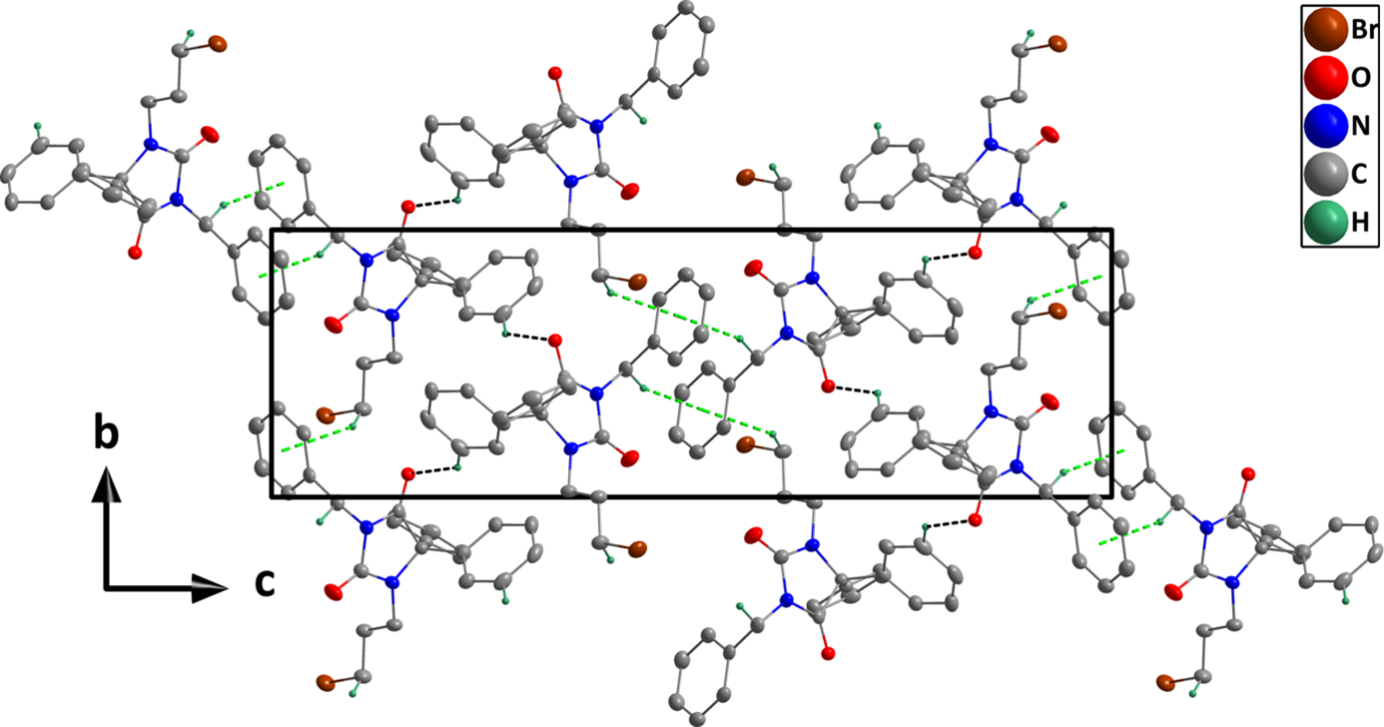
Packing viewed along the *a*-axis direction with C—H⋯O hydrogen bonds and C—H⋯π(ring) inter­actions depicted, respectively, by black and green dashed lines. Hydrogen atoms not involved in these inter­actions are omitted for clarity.

In the crystal, weak C12—H12⋯O1 hydrogen bonds and C25—H25*B*⋯*Cg*4 inter­actions (Table 1 ▸) form helical chains of mol­ecules extending along the *b*-axis direction (Fig. 2 ▸). The chains are connected by weak C16—H16*A*⋯*Cg*4 inter­actions across inversion centres (Table 1 ▸) to form the full 3-D structure (Fig. 3 ▸). Although the *D*—H⋯*A* angles in these inter­actions are noticeably less than 180°, for both C—H⋯O and C—H⋯π inter­actions, angles down to 130° have been identified as being consistent with hydrogen bond-like character for them (Steiner & Desiraju, 1998 ▸; Takahashi *et al.*, 2001 ▸).

## 4. Hirshfeld surface analysis

**Figure 4:**
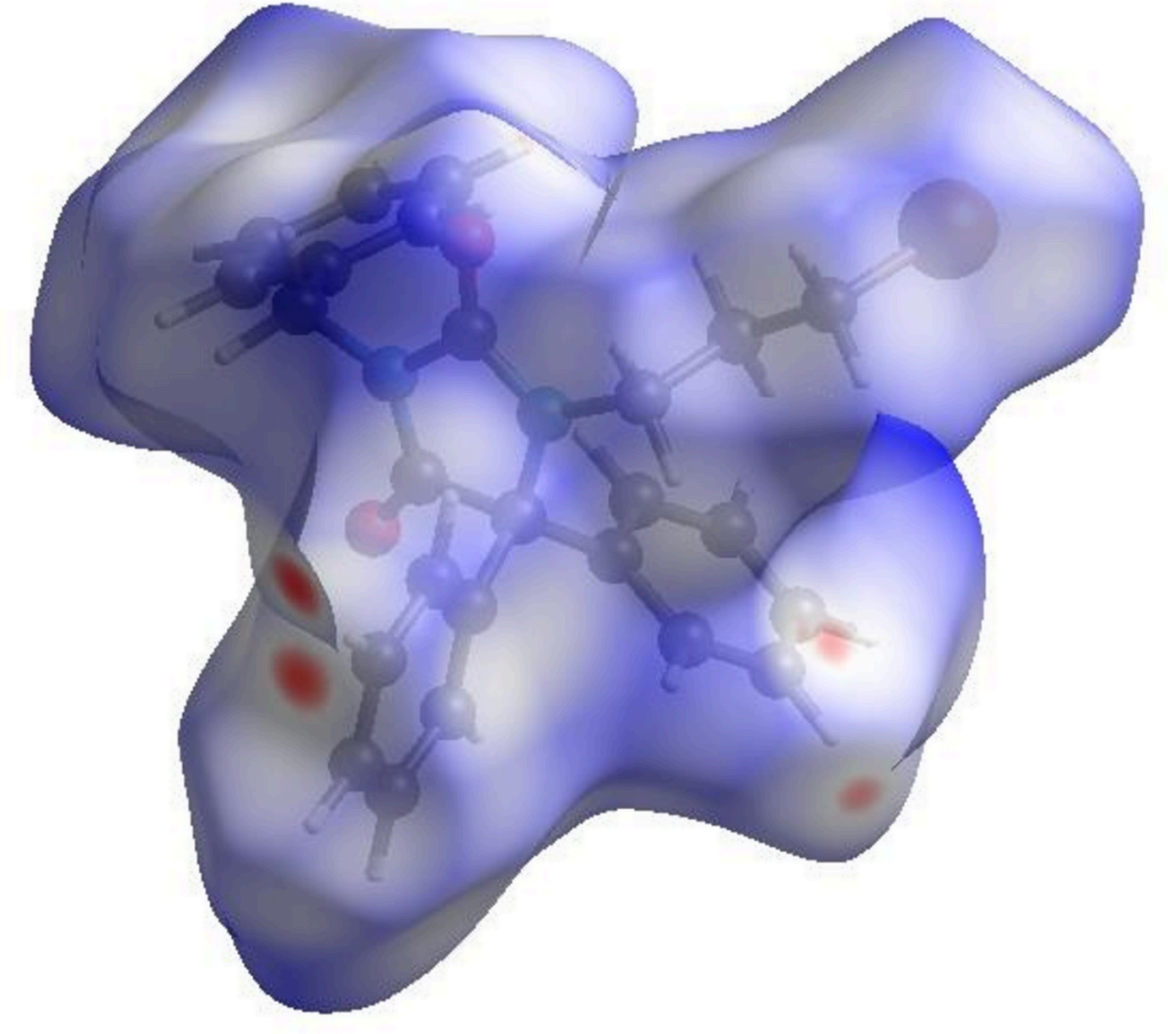
View of the three-dimensional Hirshfeld surface of the title compound plotted over *d*_norm_.

**Figure 5:**
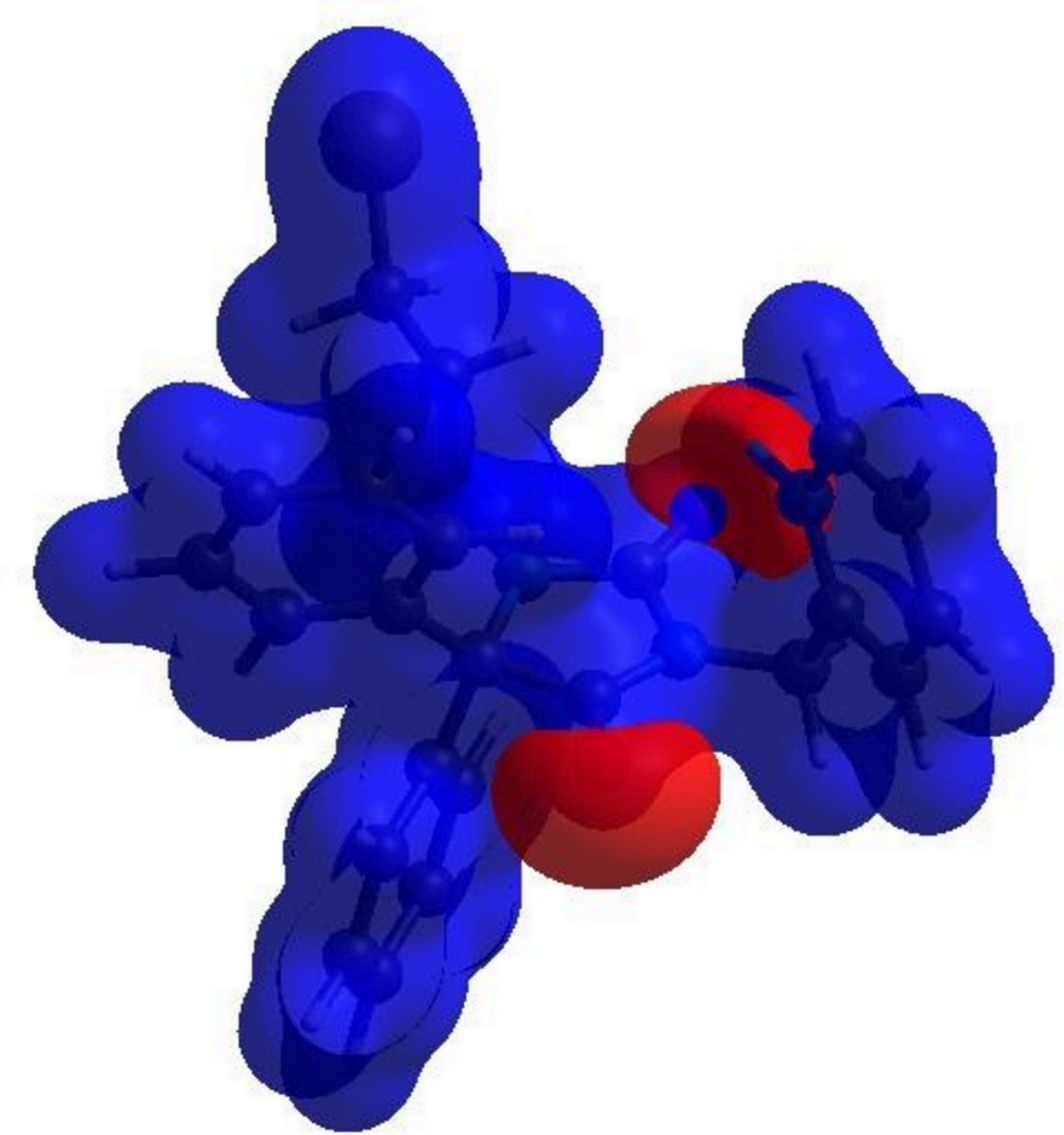
View of the ttitle compound plotted over electrostatic potential energy using the STO-3 G basis set at the Hartree–Fock level of theory. Hydrogen-bond donors and acceptors are shown as blue and red regions around the atoms corresponding to positive and negative potentials, respectively.

To visualize the inter­molecular inter­actions in the crystal, a Hirshfeld surface (HS) analysis (Hirshfeld, 1977 ▸; Spackman & Jayatilaka, 2009 ▸) was carried out by using *Crystal Explorer 17.5* (Spackman *et al.*, 2021 ▸). In the HS plotted over *d*_norm_ (Fig. 4 ▸), the white surface indicates contacts with distances equal to the sum of van der Waals radii, and the red and blue colours indicate distances shorter (in close contact) or longer (distant contact) than this sum, respectively (Venkatesan *et al.*, 2016 ▸). The bright-red spots indicate their roles as the respective donors and/or acceptors and they also appear as blue and red regions corresponding to positive (hydrogen-bond donors) and negative (hydrogen-bond acceptors) potentials on electrostatic potential plot (Spackman *et al.*, 2008 ▸; Jayatilaka *et al.*, 2005 ▸) as shown in Fig. 5 ▸.

**Figure 6:**
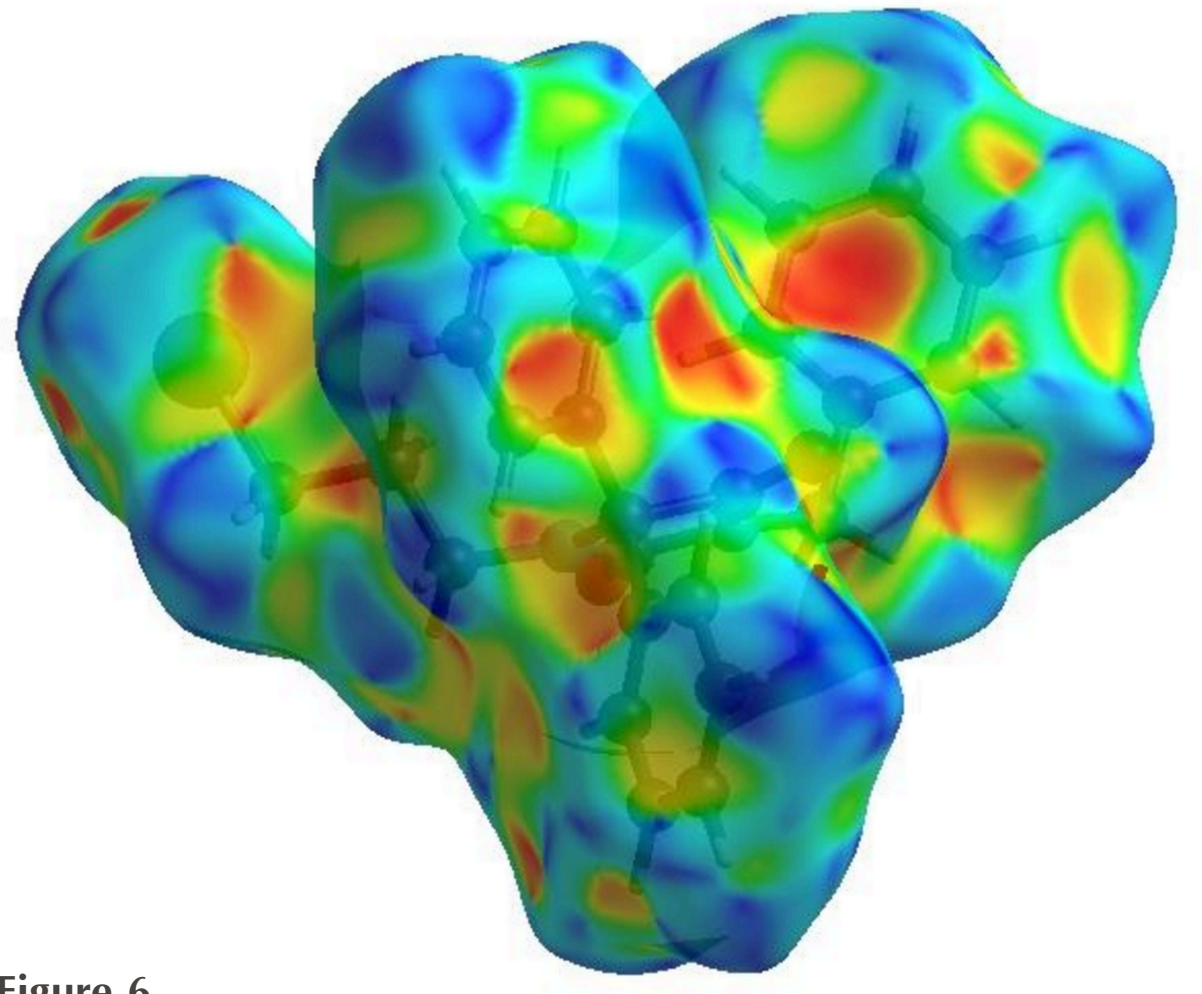
Hirshfeld surface of the title compound plotted over shape-index.

**Figure 7:**
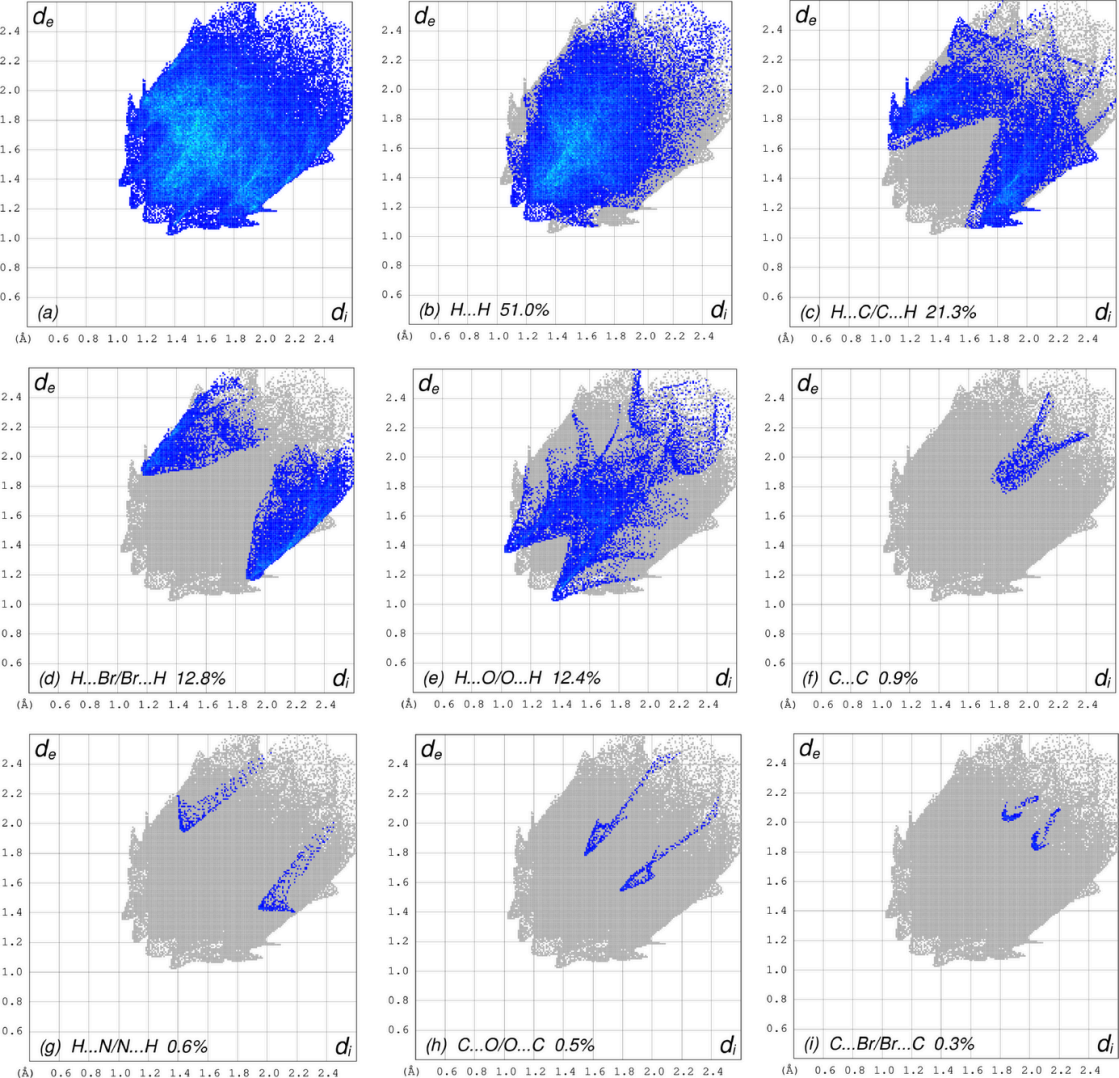
The full two-dimensional fingerprint plots for the title compound, showing (*a*) all inter­actions, and delineated into (*b*) H⋯H, (*c*) C⋯H/H⋯C (*d*) Br⋯H/H⋯Br (*e*) O⋯H/H⋯OH⋯H, C⋯H/H⋯C, Br⋯H/H⋯Br, O⋯H/H⋯O, (*f*) C⋯C, (*g*) N⋯H/H⋯N, (*h*) C⋯O/O⋯C and (i) C⋯Br/Br⋯C inter­actions. The *d*_i_ and *d*_e_ values are the closest inter­nal and external distances (in Å) from given points on the Hirshfeld surface.

Possible π–π stacking and C—H⋯π inter­actions were further visualized by plotting the surface over the shape-index. The shape-index represents the C—H⋯π inter­actions as ‘red *p*-holes’, which are related to the electron-ring inter­actions between the CH groups with the centroids of the aromatic rings of neighbouring mol­ecules. Fig. 6 ▸ clearly suggests that there are C—H⋯π inter­actions present. The presence of π–π stacking is indicated by adjacent red and blue triangles on the shape-index surface and as these are absent in Fig. 6 ▸ there are no π–π inter­actions. The overall two-dimensional fingerprint plot, Fig. 7 ▸*a*, and those delineated into H⋯H, C⋯H/H⋯C, Br⋯H/H⋯Br, O⋯H/H⋯O, C⋯C, N⋯H/H⋯N, C⋯O/O⋯C and C⋯Br/Br⋯C (McKinnon *et al.*, 2007 ▸) are illustrated in Fig. 7 ▸*b*–*i* respectively, together with their relative contributions to the Hirshfeld surface. The most important inter­action is H⋯H contributing 51.0% to the overall crystal packing, which is shown in Fig. 7 ▸*b* as widely scattered points of high density due to the large hydrogen content of the mol­ecule with the tip at *d*_e_ = *d*_i_ = 1.15 Å. The C⋯H/H⋯C contacts, contributing 21.3% to the overall crystal packing and shown in Fig. 7 ▸*c* with the tips at *d*_e_ + *d*_i_ = 2.66 Å, are mainly due to the C—H⋯π inter­actions (Table 1 ▸ and Fig. 5 ▸). The symmetrical pair of wings in the fingerprint plot delineated into Br⋯H/H⋯Br contacts (Fig. 7 ▸*d*) with the tips at *d*_e_ + *d*_i_ = 3.05 Å contributes 12.8% to the inter­molecular inter­actions. The O⋯H/H⋯O contacts, appearing as a symmetrical pair of spikes with the tips at *d*_e_ + *d*_i_ = 2.38 Å (Table 1 ▸ and Fig. 7 ▸*e*), contribute 12.4% to the total while the C⋯C (Table 2 ▸ and Fig. 7 ▸*f*), N⋯H/H⋯N (Table 3 ▸ and Fig. 7 ▸*g*), C⋯O/O⋯C (Table 2 ▸ and Fig. 7 ▸*h*) and C⋯Br/Br⋯C contacts contribute less than 1% each.

**Figure 8:**
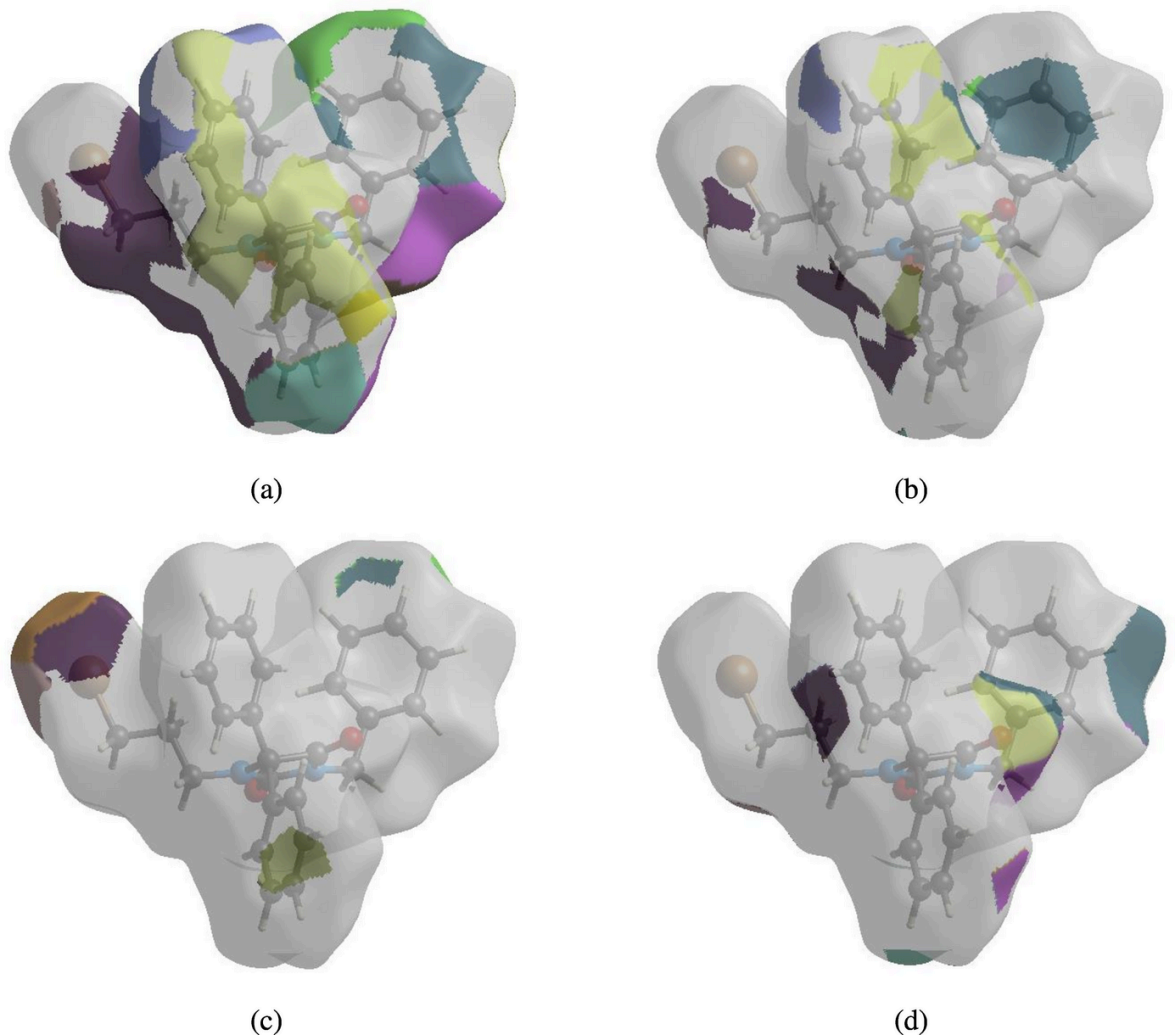
The Hirshfeld surface representations of contact patches plotted onto the surface for (*a*) H⋯H, (*b*) C⋯H/H⋯C, (*c*) Br⋯H/H⋯Br and (*d*) O⋯H/H⋯O inter­actions.

The nearest neighbour coordination environment of a mol­ecule can be determined from the colour patches on the HS based on how close to other mol­ecules they are. The Hirshfeld surface representations of contact patches plotted onto the surface are shown for the H⋯H, C⋯H/H⋯C, Br⋯H/H⋯Br, O⋯H/H⋯O inter­actions in Fig. 8 ▸*a*–*d*, respectively. These results confirm the importance of H-atom contacts in establishing the packing. The large number of H⋯H, C⋯H/H⋯C, Br⋯H/H⋯Br, O⋯H/H⋯O inter­actions suggest that van der Waals inter­actions and hydrogen bonding play the major roles in the crystal packing (Hathwar *et al.*, 2015 ▸).

## 5. Crystal voids

**Figure 9:**
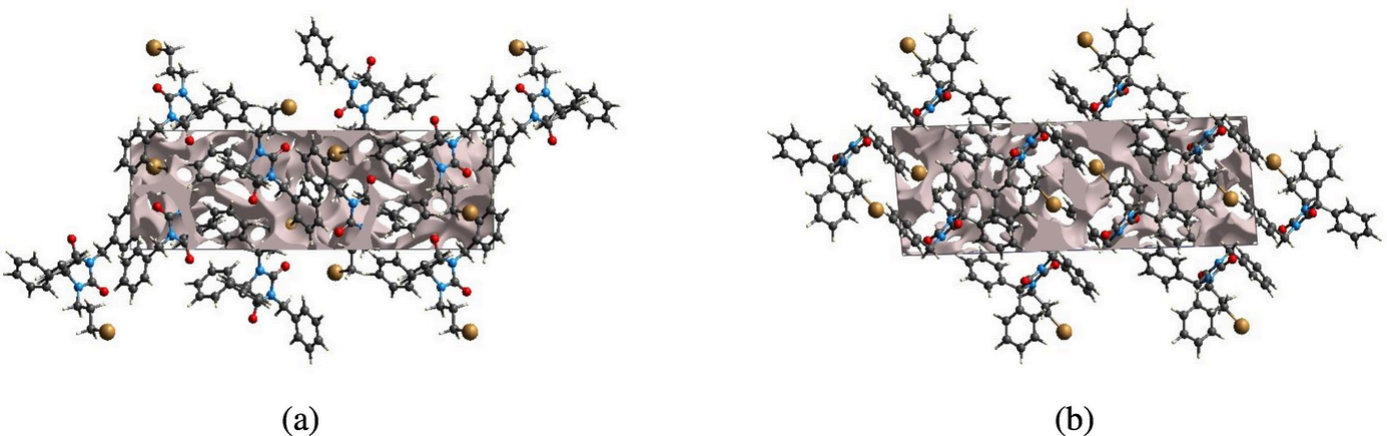
Graphical views of the voids in the crystal packing (*a*) along the *a*-axis direction and (*b*) along the *b*-axis direction.

The strength of the crystal packing is important for determining the response to an applied mechanical force. If the crystal packing results in significant voids, the mol­ecules are not tightly packed and a small amount of applied external mechanical force may easily break the crystal. To check the mechanical stability of the crystal, a void analysis was performed by adding up the electron densities of the spherically symmetric atoms contained in the asymmetric unit (Turner *et al.*, 2011 ▸). The void surface is defined as an isosurface of the procrystal electron density and is calculated for the whole unit cell where the void surface meets the boundary of the unit cell and capping faces are generated to create an enclosed volume. The volume of the crystal voids (Fig. 9 ▸*a* and *b*) and the percentage of free space in the unit cell are calculated as 251.24 Å^3^ and 11.71%, respectively. Thus, the crystal packing appears compact and the mechanical stability should be substantial.

## 6. Inter­action energy calculations and energy frameworks

**Figure 10:**
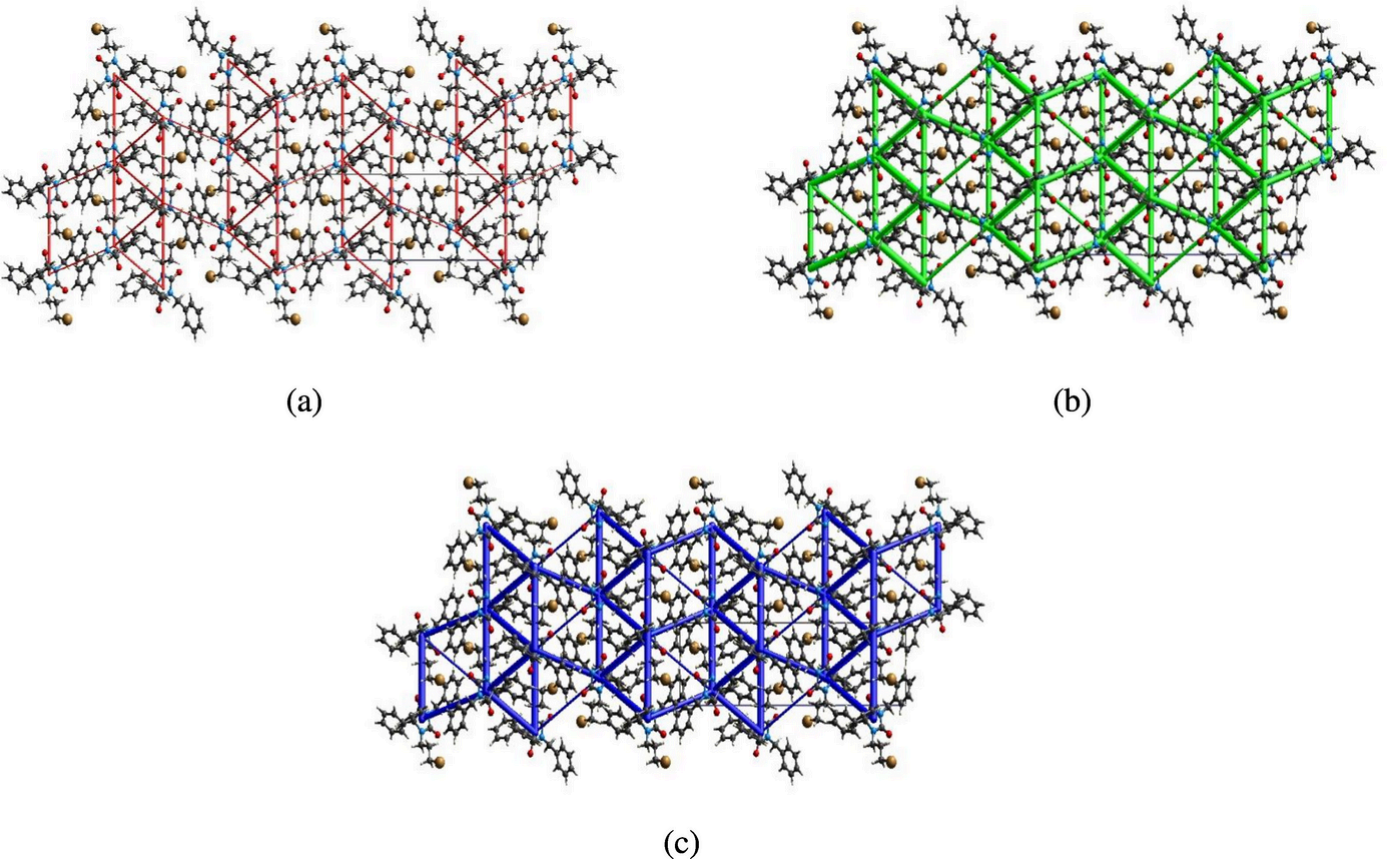
The energy frameworks for a cluster of mol­ecules of the title compound viewed down the *a*-axis direction showing (*a*) electrostatic energy, (*b*) dispersion energy and (*c*) total energy diagrams. The cylindrical radius is proportional to the relative strength of the corresponding energies and they were adjusted to the same scale factor of 80 with a cut-off value of 5 kJ mol^−1^ within 2 × 2 × 2 unit cells.

The inter­molecular inter­action energies were calculated using the CE–HF/3–21G energy model available in *Crystal Explorer 17.5* (Spackman *et al.*, 2021 ▸), where a cluster of mol­ecules is generated by applying crystallographic symmetry operations with respect to a selected central mol­ecule within the radius of 3.8 Å (Turner *et al.*, 2014 ▸). The total inter­molecular energy (*E*_tot_) is the sum of electrostatic (*E*_ele_), polarization (*E*_pol_), dispersion (*E*_dis_) and exchange-repulsion (*E*_rep_) energies (Turner *et al.*, 2015 ▸) with scale factors of 1.019, 0.651, 0.901 and 0.811, respectively (Mackenzie *et al.*, 2017 ▸). Hydrogen-bonding inter­action energies (in kJ mol^−1^) were calculated to be −22.9 (*E*_ele_), −7.5 (*E*_pol_), −42.9 (*E*_dis_), 18.5 (*E*_rep_) and −54.8 (*E*_tot_) for the C12—H12⋯O1 hydrogen-bonding inter­action. Energy frameworks combine the calculation of inter­molecular inter­action energies with a graphical representation of their magnitude (Turner *et al.*, 2015 ▸). Energies between mol­ecular pairs are represented as cylinders joining the centroids of pairs of mol­ecules with the cylinder radius proportional to the relative strength of the corresponding inter­action energy. Energy frameworks were constructed for *E*_ele_ (red cylinders), *E*_dis_ (green cylinders) and *E*_tot_ (blue cylinders) (Fig. 10 ▸*a*, *b* and *c*). The evaluation of the electrostatic, dispersion and total energy frameworks indicate that the stabilization is dominated by the dispersion energy contributions in the crystal structure.

## 7. Database survey

**Figure 11:**
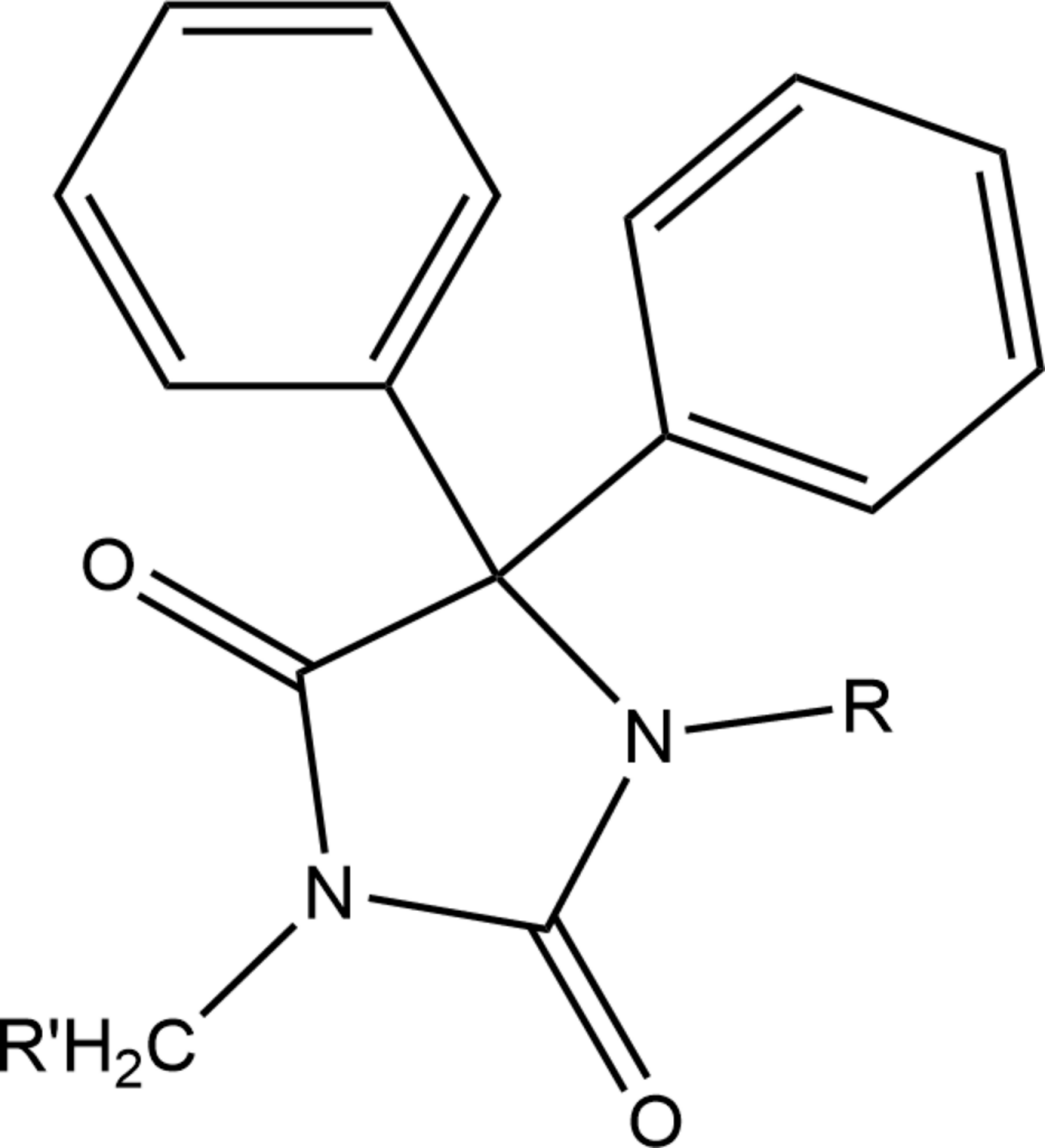
Fragment used for the database search.

A survey of the Cambridge Structural Database (CSD, updated to June 2024; Groom *et al.*, 2016 ▸) with the search fragment shown in Fig. 11 ▸ (*R* = *R*′ = nothing) yielded 37 hits, of which 15 were considered not similar to the title mol­ecule as they were either ionic or were spiro­(fluorene-9,5-imidazolidin)-2,4-dione derivatives instead of having two independent phenyl groups. Of the remaining 22, eighteen had *R* = H with *R*′ = allyl (BUCDEL; Guerrab *et al.*, 2020*a* ▸), CH_2_CH(OH)CH_2_N(CH_2_CH_2_)_2_NPh (EKANOT; Kieć-Kononowicz *et al.*, 2003 ▸), Et (Guerrab *et al.*, 2017*a* ▸), *n*-pentyl (GEMSOJ; Guerrab *et al.*, 2017*b* ▸), CH_2_C(=O)(4-FC_6_H_4_) (GITSOT; Mague *et al.*, 2014 ▸, GITSOT01; Alnazi *et al.*, 2013 ▸), CH_2_COOEt (JALGEL; Ramli, *et al.*, 2017 ▸), benzyl (MESSAH; Guerrab *et al.*, 2018 ▸), CH_2_CH2Br (NIBMOE; Guerrab *et al.*, 2023 ▸), *n*-decyl (PAJMAS; Guerrab *et al.*, 2021 ▸), Me (PEPDUM; Guerrab *et al.*, 2017 ▸*c*), *n*-octyl (QAGPAT; Guerrab *et al.*, 2020*b* ▸), *n*-butyl (QUNBET; Guerrab *et al.*, 2018*b* ▸), *n*-hexyl (QENBOD; Guerrab *et al.*, 2018*c* ▸), *n*-propyl (WEMQUD; Guerrab *et al.*, 2017*d* ▸. WEMQUD01; Trišović *et al.*, 2019 ▸), *i*-butyl TEDYOZ; Guerrab *et al.*, 2022 ▸) and CH_2_CH_2_N(CH_2_CH_2_)_2_O (LOKXAO; Lamssane *et al.*, 2024 ▸). In most of these, the five-membered rings are somewhat ruffled with deviations of atoms from the mean plane of up to 0.053 (2) Å except for FEHPUG and QENBET where the largest deviations were only 0.006 (1) and 0.005 (1) Å, respectively. The minimum and maximum dihedral angles between the mean plane of the five-membered ring and an attached phenyl ring are 53.21 (1) and 84.94 (16)°, respectively, and the difference between these dihedral angles in a given mol­ecule ranged from essentially 0° (MESSAH) to about 24° (EKANOT). The main determinant of the supra­molecular structures is N—H⋯O hydrogen bonds, which either form chains of mol­ecules or inversion dimers. These are further connected by C—H⋯O hydrogen bonds and C—H⋯π(ring) inter­actions with the detailed, 3-D structures influenced by the nature and bulk of *R*′. The four mol­ecules with substituents on both nitro­gen atoms of the five-membered ring have *R* = CH_2_CH(OH)CH_2_NH^*i*^Pr and *R*′ = CH_2_COOMe (EKANIN; Kieć-Kononowicz *et al.*, 2003 ▸), *R* = benzyl and *R*′ = CH_2_COOH (HAVLOF; Ciechanowicz-Rutkowska *et al.*, 1994 ▸), *R* = CH_2_COOH and *R*′ = CH_2_(2,4-Cl_2_C_6_H_3_) (HAVLUL; Ciechanowicz-Rutkowska *et al.*, 1994 ▸) and *R* = *R*′ = CH_2_C≡CH (XOLLUI; Ghandour *et al.*, 2019 ▸). In these, the five-membered rings are somewhat more ruffled than in the previous group and the dihedral angles between the five-membered ring and the attached phenyl groups range from 64.30 (17)° (HAVLUL) to 82.8 (4)° (EKANIN). The first three contain OH groups in the side chain so O—H⋯O hydrogen bonds are the dominant packing inter­action and, again, either chains or dimers are formed from these. These units are further linked by C—H⋯O hydrogen bonds and C—H⋯π(ring) inter­actions. In XOLLUI, C—H⋯O hydrogen bonds and weak C—H⋯π(ring) inter­actions generate the supra­molecular structure.

## 8. Synthesis and crystallization

In a flask, 3-benzyl-5,5-di­phenyl­imidazolidine-2,4-dione (0.5 g, 1.46 mmol) was dissolved in 20 mL of acetone. Potassium carbonate, (K_2_CO_3_; 0.3 g, 2.17 mmol) and tetra-*n*-butyl­ammonium bromide (BTBA; 0.05 g, 0.14 mmol) were added and the mixture was stirred for 30 min. After that, 1,3-di­bromo­propane (0.35 g, 1.73 mmol) was added and the mixture was stirred at ambient temperature for 48 h. The solvent was evaporated under reduced pressure and the salts removed by liquid–liquid extraction with water and di­chloro­methane. The resulting residue was purified using silica column chromatography with an ethyl acetate/hexane (1/6) solvent system and recrystallized from ethanol. Yield 68%. Colourless crystals. *R*_f_: 0.54 (ethyl acetate/hexa­ne: 1/4), m.p. 378–380 K. LCMS (ESI): 463.10147 [*M* + H^+^]. ^1^H NMR (CDCl_3_, 600.13 MHz): δ (ppm) 7.22–7.42 (*m*, 15H, HAr); 4.76 (*s*, 1H, CH_2_), 3.53 (*t*, 2H, CH_2_, ^3^*J*_H–H_ = 6 Hz), 3.10 (*t*, 2H, CH_2_, ^3^*J*_H–H_ = 6 Hz), 1.45 (*qt*, 2H, CH_2_, ^3^*J*_H–H_ = 6 Hz). ^13^C NMR (CDCl_3_, 100.62 MHz): δ (ppm) 173.35, 155.74 (C=O); 136.90, 136.07, 74.93 (Cq); 127.95–129.08 (CHAr); 42.92, 41.00, 30.57, 30.48 (CH_2_).

## 9. Refinement

Crystal data, data collection and structure refinement details are summarized in Table 3 ▸. H atoms attached to carbon were placed in calculated positions (C—H = 0.95–0.99 Å) and included as riding contributions with isotropic displacement parameters 1.2–1.5 times those of the parent atoms.

## Supplementary Material

Crystal structure: contains datablock(s) global, I. DOI: 10.1107/S2056989024009228/jy2051sup1.cif

Supporting information file. DOI: 10.1107/S2056989024009228/jy2051Isup3.cdx

Structure factors: contains datablock(s) I. DOI: 10.1107/S2056989024009228/jy2051Isup4.hkl

Supporting information file. DOI: 10.1107/S2056989024009228/jy2051Isup4.cml

CCDC reference: 2385417

Additional supporting information:  crystallographic information; 3D view; checkCIF report

## Figures and Tables

**Table 1:** Hydrogen-bond geometry (Å, °) *Cg*4 is the centroid of the C17–C22 benzene ring.

*D*—H⋯*A*	*D*—H	H⋯*A*	*D*⋯*A*	*D*—H⋯*A*
C12—H12⋯O1^i^	0.95	2.49	3.2158 (17)	133
C16—H16*A*⋯*Cg*4^v^	0.99	2.66	3.5901 (14)	157
C25—H25*B*⋯*Cg*4^vi^	0.99	2.89	3.7621 (17)	148

Symmetry codes: (i) 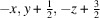; (v) 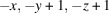; (vi) 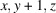.

**Table 2:** Selected interatomic distances (Å)

O1⋯C5	3.2074 (16)	C2⋯H5	2.57
O1⋯C15	3.2170 (16)	C4⋯H15	2.76
H12⋯O1^i^	2.49	C4⋯H24*A*	2.90
H8⋯O1^ii^	2.62	C9⋯H15	2.80
H21⋯O2^iii^	2.63	C10⋯H9	2.65
O2⋯H23*B*	2.66	C12⋯H8^iv^	2.88
O2⋯H16*A*	2.56	C15⋯H9	2.73
N1⋯H18	2.75	C17⋯H16*A*^v^	2.90
N2⋯H11	2.45	C23⋯H11	2.87
C9⋯C15	3.1165 (19)	H16*B*⋯H22	2.36
C11⋯C23	3.3928 (19)		

Symmetry codes: (i) 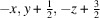; (ii) 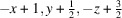; (iii) 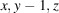; (iv) 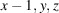; (v) 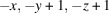.

**Table 3:** Experimental details

Crystal data	
Chemical formula	C_25_H_23_BrN_2_O_2_
*M*_r_	463.36
Crystal system, space group	Monoclinic, *P*2_1_/*c*
Temperature (K)	150
*a*, *b*, *c* (Å)	9.4306 (3), 8.5084 (3), 26.7891 (10)
β (°)	93.270 (1)
*V* (Å^3^)	2146.04 (13)
*Z*	4
Radiation type	Cu *K*α
μ (mm^−1^)	2.80
Crystal size (mm)	0.13 × 0.11 × 0.11

Data collection	
Diffractometer	Bruker D8 VENTURE PHOTON 3 CPAD
Absorption correction	Multi-scan (*SADABS*; Krause *et al.*, 2015 ▸)
*T*_min_, *T*_max_	0.69, 0.75
No. of measured, independent and observed [*I* > 2σ(*I*)] reflections	87214, 4348, 4262
*R*_int_	0.028
(sin θ/λ)_max_ (Å^−1^)	0.625

Refinement	
*R*[*F*^2^ > 2σ(*F*^2^)], *wR*(*F*^2^), *S*	0.024, 0.060, 1.05
No. of reflections	4348
No. of parameters	271
H-atom treatment	H-atom parameters constrained
Δρ_max_, Δρ_min_ (e Å^−3^)	0.35, −0.39

Computer programs: *APEX4* and *SAINT* (Bruker, 2021 ▸), *SHELXT2015* (Sheldrick, 2015*a* ▸), *SHELXL* (Sheldrick, 2015*b* ▸), *DIAMOND* (Brandenburg & Putz, 2012 ▸) and *SHELXTL* (Sheldrick, 2008 ▸).

## supplementary crystallographic information

### 3-Benzyl-1-(3-bromopropyl)-5,5-diphenylimidazolidine-2,4-dione Crystal data

**Table:**

C_25_H_23_BrN_2_O_2_	*F*(000) = 952
*M**_r_* = 463.36	*D*_x_ = 1.434 Mg m^−^^3^
Monoclinic, *P*2_1_/*c*	Cu *K*α radiation, λ = 1.54178 Å
*a* = 9.4306 (3) Å	Cell parameters from 9871 reflections
*b* = 8.5084 (3) Å	θ = 3.3–74.6°
*c* = 26.7891 (10) Å	µ = 2.80 mm^−^^1^
β = 93.270 (1)°	*T* = 150 K
*V* = 2146.04 (13) Å^3^	Block, colourless
*Z* = 4	0.13 × 0.11 × 0.11 mm

### 3-Benzyl-1-(3-bromopropyl)-5,5-diphenylimidazolidine-2,4-dione Data collection

**Table:**

Bruker D8 VENTURE PHOTON 3 CPAD diffractometer	4348 independent reflections
Radiation source: INCOATEC IµS micro—-focus source	4262 reflections with *I* > 2σ(*I*)
Mirror monochromator	*R*_int_ = 0.028
Detector resolution: 7.3910 pixels mm^-1^	θ_max_ = 74.6°, θ_min_ = 3.3°
φ and ω scans	*h* = −11→11
Absorption correction: multi-scan (*SADABS*; Krause *et al.*, 2015)	*k* = −10→10
*T*_min_ = 0.69, *T*_max_ = 0.75	*l* = −33→33
87214 measured reflections	

### 3-Benzyl-1-(3-bromopropyl)-5,5-diphenylimidazolidine-2,4-dione Refinement

**Table:**

Refinement on *F*^2^	Primary atom site location: dual
Least-squares matrix: full	Secondary atom site location: difference Fourier map
*R*[*F*^2^ > 2σ(*F*^2^)] = 0.024	Hydrogen site location: inferred from neighbouring sites
*wR*(*F*^2^) = 0.060	H-atom parameters constrained
*S* = 1.05	*w* = 1/[σ^2^(*F*_o_^2^) + (0.0239*P*)^2^ + 1.2411*P*] where *P* = (*F*_o_^2^ + 2*F*_c_^2^)/3
4348 reflections	(Δ/σ)_max_ = 0.004
271 parameters	Δρ_max_ = 0.35 e Å^−^^3^
0 restraints	Δρ_min_ = −0.39 e Å^−^^3^

### 3-Benzyl-1-(3-bromopropyl)-5,5-diphenylimidazolidine-2,4-dione Special details

**Table:**

Experimental. The diffraction data were obtained from 30 sets of frames, each of width 0.5° in ω or φ, collected with scan parameters determined by the "strategy" routine in *APEX4*. The scan time was θ-dependent and ranged from 2 to 10 sec/frame.
Geometry. All esds (except the esd in the dihedral angle between two l.s. planes) are estimated using the full covariance matrix. The cell esds are taken into account individually in the estimation of esds in distances, angles and torsion angles; correlations between esds in cell parameters are only used when they are defined by crystal symmetry. An approximate (isotropic) treatment of cell esds is used for estimating esds involving l.s. planes.
Refinement. Refinement of F^2^ against ALL reflections. The weighted R-factor wR and goodness of fit S are based on F^2^, conventional R-factors R are based on F, with F set to zero for negative F^2^. The threshold expression of F^2^ > 2sigma(F^2^) is used only for calculating R-factors(gt) etc. and is not relevant to the choice of reflections for refinement. R-factors based on F^2^ are statistically about twice as large as those based on F, and R- factors based on ALL data will be even larger. H-atoms attached to carbon were placed in calculated positions (C—H = 0.95 - 0.99 Å). All were included as riding contributions with isotropic displacement parameters 1.2 - 1.5 times those of the attached atoms.

### 3-Benzyl-1-(3-bromopropyl)-5,5-diphenylimidazolidine-2,4-dione Fractional atomic coordinates and isotropic or equivalent isotropic displacement parameters (Å^2^)

**Table:**

	*x*	*y*	*z*	*U*_iso_*/*U*_eq_	
Br1	0.63895 (2)	1.19589 (2)	0.56429 (2)	0.03559 (6)	
O1	0.23072 (9)	0.41429 (10)	0.66257 (3)	0.02231 (18)	
O2	0.10937 (12)	0.85648 (13)	0.57357 (4)	0.0371 (2)	
N1	0.13721 (11)	0.61386 (13)	0.61309 (4)	0.0222 (2)	
N2	0.25962 (11)	0.81958 (12)	0.64354 (4)	0.0218 (2)	
C1	0.29725 (13)	0.69120 (14)	0.67837 (4)	0.0189 (2)	
C2	0.22121 (12)	0.55173 (15)	0.65126 (4)	0.0189 (2)	
C3	0.16363 (14)	0.77601 (16)	0.60666 (5)	0.0249 (3)	
C4	0.45740 (13)	0.66583 (14)	0.68382 (5)	0.0198 (2)	
C5	0.52555 (14)	0.58925 (16)	0.64591 (5)	0.0256 (3)	
H5	0.471139	0.548166	0.617953	0.031*	
C6	0.67213 (15)	0.57249 (18)	0.64864 (6)	0.0320 (3)	
H6	0.717723	0.520761	0.622523	0.038*	
C7	0.75206 (14)	0.63127 (17)	0.68951 (6)	0.0314 (3)	
H7	0.852430	0.619850	0.691417	0.038*	
C8	0.68546 (15)	0.70657 (16)	0.72751 (5)	0.0286 (3)	
H8	0.740099	0.745919	0.755670	0.034*	
C9	0.53839 (14)	0.72480 (16)	0.72455 (5)	0.0243 (3)	
H9	0.493245	0.777830	0.750515	0.029*	
C10	0.22387 (13)	0.70770 (14)	0.72776 (5)	0.0206 (2)	
C11	0.11328 (14)	0.81385 (16)	0.73237 (5)	0.0260 (3)	
H11	0.089411	0.885625	0.706059	0.031*	
C12	0.03746 (15)	0.81517 (18)	0.77547 (6)	0.0315 (3)	
H12	−0.037298	0.888758	0.778555	0.038*	
C13	0.07011 (16)	0.71037 (18)	0.81373 (5)	0.0331 (3)	
H13	0.017278	0.710944	0.842878	0.040*	
C14	0.18033 (16)	0.60422 (18)	0.80946 (5)	0.0312 (3)	
H14	0.203268	0.532104	0.835765	0.037*	
C15	0.25732 (14)	0.60323 (16)	0.76670 (5)	0.0252 (3)	
H15	0.333213	0.530848	0.764033	0.030*	
C16	0.04615 (13)	0.52076 (16)	0.57837 (5)	0.0249 (3)	
H16A	−0.014958	0.592296	0.557440	0.030*	
H16B	−0.016421	0.453160	0.597633	0.030*	
C17	0.13128 (14)	0.41896 (16)	0.54494 (5)	0.0235 (3)	
C18	0.24295 (15)	0.48328 (17)	0.51974 (5)	0.0276 (3)	
H18	0.265958	0.591359	0.523930	0.033*	
C19	0.32050 (16)	0.39077 (19)	0.48868 (5)	0.0323 (3)	
H19	0.396777	0.435428	0.471840	0.039*	
C20	0.28716 (17)	0.23303 (19)	0.48208 (6)	0.0346 (3)	
H20	0.340292	0.169796	0.460658	0.042*	
C21	0.17633 (18)	0.16807 (18)	0.50677 (5)	0.0342 (3)	
H21	0.153035	0.060211	0.502189	0.041*	
C22	0.09907 (15)	0.26050 (17)	0.53826 (5)	0.0292 (3)	
H22	0.023634	0.215106	0.555364	0.035*	
C23	0.31763 (14)	0.97844 (15)	0.64620 (5)	0.0250 (3)	
H23A	0.354179	1.000408	0.680878	0.030*	
H23B	0.241029	1.054855	0.637461	0.030*	
C24	0.43793 (14)	0.99988 (15)	0.61061 (5)	0.0269 (3)	
H24A	0.520223	0.934448	0.622133	0.032*	
H24B	0.405496	0.964347	0.576670	0.032*	
C25	0.48288 (16)	1.17047 (16)	0.60868 (6)	0.0298 (3)	
H25A	0.513553	1.206741	0.642713	0.036*	
H25B	0.401208	1.235731	0.596448	0.036*	

### 3-Benzyl-1-(3-bromopropyl)-5,5-diphenylimidazolidine-2,4-dione Atomic displacement parameters (Å^2^)

**Table:**

	*U*^11^	*U*^22^	*U*^33^	*U*^12^	*U*^13^	*U*^23^
Br1	0.03717 (10)	0.03173 (10)	0.03858 (10)	−0.00687 (6)	0.00825 (7)	0.00692 (6)
O1	0.0252 (4)	0.0202 (4)	0.0217 (4)	−0.0023 (3)	0.0024 (3)	0.0009 (3)
O2	0.0411 (6)	0.0316 (5)	0.0369 (6)	−0.0024 (5)	−0.0134 (5)	0.0119 (5)
N1	0.0225 (5)	0.0231 (5)	0.0205 (5)	−0.0024 (4)	−0.0028 (4)	0.0013 (4)
N2	0.0230 (5)	0.0189 (5)	0.0232 (5)	−0.0012 (4)	−0.0008 (4)	0.0031 (4)
C1	0.0193 (6)	0.0180 (6)	0.0193 (6)	−0.0002 (4)	0.0007 (4)	0.0001 (4)
C2	0.0174 (5)	0.0225 (6)	0.0171 (5)	−0.0013 (5)	0.0039 (4)	−0.0006 (5)
C3	0.0243 (6)	0.0246 (6)	0.0257 (6)	−0.0009 (5)	0.0004 (5)	0.0029 (5)
C4	0.0194 (6)	0.0178 (5)	0.0223 (6)	−0.0008 (4)	0.0012 (4)	0.0022 (5)
C5	0.0226 (6)	0.0276 (7)	0.0264 (6)	−0.0003 (5)	0.0010 (5)	−0.0044 (5)
C6	0.0242 (7)	0.0330 (7)	0.0391 (8)	0.0035 (6)	0.0060 (6)	−0.0065 (6)
C7	0.0195 (6)	0.0284 (7)	0.0459 (8)	0.0013 (5)	−0.0011 (6)	0.0017 (6)
C8	0.0245 (7)	0.0278 (7)	0.0328 (7)	−0.0044 (5)	−0.0061 (5)	0.0016 (5)
C9	0.0244 (6)	0.0232 (6)	0.0253 (6)	−0.0023 (5)	0.0004 (5)	−0.0016 (5)
C10	0.0192 (6)	0.0218 (6)	0.0209 (6)	−0.0030 (5)	0.0012 (5)	−0.0045 (5)
C11	0.0212 (6)	0.0270 (7)	0.0297 (7)	−0.0002 (5)	0.0009 (5)	−0.0047 (5)
C12	0.0213 (6)	0.0356 (8)	0.0381 (8)	−0.0021 (5)	0.0067 (6)	−0.0137 (6)
C13	0.0302 (7)	0.0425 (8)	0.0277 (7)	−0.0122 (6)	0.0106 (6)	−0.0134 (6)
C14	0.0364 (7)	0.0369 (8)	0.0207 (6)	−0.0091 (6)	0.0030 (5)	−0.0016 (6)
C15	0.0262 (6)	0.0265 (7)	0.0231 (6)	−0.0011 (5)	0.0017 (5)	−0.0017 (5)
C16	0.0224 (6)	0.0296 (7)	0.0223 (6)	−0.0056 (5)	−0.0037 (5)	0.0000 (5)
C17	0.0250 (6)	0.0273 (7)	0.0177 (6)	−0.0035 (5)	−0.0050 (5)	0.0026 (5)
C18	0.0311 (7)	0.0268 (7)	0.0247 (6)	−0.0063 (5)	−0.0002 (5)	0.0011 (5)
C19	0.0314 (7)	0.0376 (8)	0.0280 (7)	−0.0041 (6)	0.0035 (5)	0.0012 (6)
C20	0.0405 (8)	0.0342 (8)	0.0290 (7)	0.0056 (6)	0.0001 (6)	−0.0024 (6)
C21	0.0472 (9)	0.0244 (7)	0.0302 (7)	−0.0019 (6)	−0.0040 (6)	0.0010 (6)
C22	0.0346 (7)	0.0284 (7)	0.0240 (6)	−0.0077 (6)	−0.0029 (5)	0.0036 (5)
C23	0.0270 (6)	0.0181 (6)	0.0300 (6)	−0.0008 (5)	0.0021 (5)	−0.0001 (5)
C24	0.0290 (7)	0.0205 (6)	0.0316 (7)	−0.0018 (5)	0.0048 (5)	0.0009 (5)
C25	0.0322 (7)	0.0231 (7)	0.0346 (7)	−0.0029 (5)	0.0063 (6)	0.0024 (6)

### 3-Benzyl-1-(3-bromopropyl)-5,5-diphenylimidazolidine-2,4-dione Geometric parameters (Å, º)

**Table:**

Br1—C25	1.9562 (14)	C12—H12	0.9500
O1—C2	1.2100 (15)	C13—C14	1.387 (2)
O2—C3	1.2103 (17)	C13—H13	0.9500
N1—C2	1.3639 (16)	C14—C15	1.3908 (18)
N1—C3	1.4142 (17)	C14—H14	0.9500
N1—C16	1.4626 (16)	C15—H15	0.9500
N2—C3	1.3530 (17)	C16—C17	1.5095 (19)
N2—C23	1.4584 (16)	C16—H16A	0.9900
N2—C1	1.4669 (15)	C16—H16B	0.9900
C1—C4	1.5244 (16)	C17—C22	1.3914 (19)
C1—C10	1.5343 (17)	C17—C18	1.3947 (19)
C1—C2	1.5461 (16)	C18—C19	1.384 (2)
C4—C9	1.3895 (18)	C18—H18	0.9500
C4—C5	1.3940 (18)	C19—C20	1.388 (2)
C5—C6	1.3874 (19)	C19—H19	0.9500
C5—H5	0.9500	C20—C21	1.384 (2)
C6—C7	1.387 (2)	C20—H20	0.9500
C6—H6	0.9500	C21—C22	1.390 (2)
C7—C8	1.383 (2)	C21—H21	0.9500
C7—H7	0.9500	C22—H22	0.9500
C8—C9	1.3934 (19)	C23—C24	1.5339 (18)
C8—H8	0.9500	C23—H23A	0.9900
C9—H9	0.9500	C23—H23B	0.9900
C10—C11	1.3904 (18)	C24—C25	1.5138 (18)
C10—C15	1.3928 (18)	C24—H24A	0.9900
C11—C12	1.392 (2)	C24—H24B	0.9900
C11—H11	0.9500	C25—H25A	0.9900
C12—C13	1.380 (2)	C25—H25B	0.9900
			
O1···C5	3.2074 (16)	C2···H5	2.57
O1···C15	3.2170 (16)	C4···H15	2.76
H12···O1^i^	2.49	C4···H24A	2.90
H8···O1^ii^	2.62	C9···H15	2.80
H21···O2^iii^	2.63	C10···H9	2.65
O2···H23B	2.66	C12···H8^iv^	2.88
O2···H16A	2.56	C15···H9	2.73
N1···H18	2.75	C17···H16A^v^	2.90
N2···H11	2.45	C23···H11	2.87
C9···C15	3.1165 (19)	H16B···H22	2.36
C11···C23	3.3928 (19)		
			
C2—N1—C3	111.75 (10)	C13—C14—C15	120.06 (14)
C2—N1—C16	124.18 (11)	C13—C14—H14	120.0
C3—N1—C16	123.51 (11)	C15—C14—H14	120.0
C3—N2—C23	121.54 (11)	C14—C15—C10	120.42 (13)
C3—N2—C1	112.83 (10)	C14—C15—H15	119.8
C23—N2—C1	125.63 (10)	C10—C15—H15	119.8
N2—C1—C4	111.64 (10)	N1—C16—C17	112.04 (10)
N2—C1—C10	112.08 (10)	N1—C16—H16A	109.2
C4—C1—C10	115.06 (10)	C17—C16—H16A	109.2
N2—C1—C2	100.59 (9)	N1—C16—H16B	109.2
C4—C1—C2	111.51 (10)	C17—C16—H16B	109.2
C10—C1—C2	104.80 (9)	H16A—C16—H16B	107.9
O1—C2—N1	126.43 (11)	C22—C17—C18	118.90 (13)
O1—C2—C1	126.76 (11)	C22—C17—C16	120.85 (12)
N1—C2—C1	106.78 (10)	C18—C17—C16	120.25 (12)
O2—C3—N2	128.11 (13)	C19—C18—C17	120.48 (13)
O2—C3—N1	124.82 (12)	C19—C18—H18	119.8
N2—C3—N1	107.07 (11)	C17—C18—H18	119.8
C9—C4—C5	119.03 (12)	C18—C19—C20	120.20 (14)
C9—C4—C1	121.41 (11)	C18—C19—H19	119.9
C5—C4—C1	119.47 (11)	C20—C19—H19	119.9
C6—C5—C4	120.59 (12)	C21—C20—C19	119.82 (14)
C6—C5—H5	119.7	C21—C20—H20	120.1
C4—C5—H5	119.7	C19—C20—H20	120.1
C7—C6—C5	119.98 (13)	C20—C21—C22	120.05 (14)
C7—C6—H6	120.0	C20—C21—H21	120.0
C5—C6—H6	120.0	C22—C21—H21	120.0
C8—C7—C6	119.92 (13)	C21—C22—C17	120.55 (13)
C8—C7—H7	120.0	C21—C22—H22	119.7
C6—C7—H7	120.0	C17—C22—H22	119.7
C7—C8—C9	120.13 (13)	N2—C23—C24	111.62 (11)
C7—C8—H8	119.9	N2—C23—H23A	109.3
C9—C8—H8	119.9	C24—C23—H23A	109.3
C4—C9—C8	120.35 (12)	N2—C23—H23B	109.3
C4—C9—H9	119.8	C24—C23—H23B	109.3
C8—C9—H9	119.8	H23A—C23—H23B	108.0
C11—C10—C15	119.14 (12)	C25—C24—C23	110.72 (11)
C11—C10—C1	120.90 (11)	C25—C24—H24A	109.5
C15—C10—C1	119.57 (11)	C23—C24—H24A	109.5
C10—C11—C12	120.15 (13)	C25—C24—H24B	109.5
C10—C11—H11	119.9	C23—C24—H24B	109.5
C12—C11—H11	119.9	H24A—C24—H24B	108.1
C13—C12—C11	120.48 (13)	C24—C25—Br1	110.41 (9)
C13—C12—H12	119.8	C24—C25—H25A	109.6
C11—C12—H12	119.8	Br1—C25—H25A	109.6
C12—C13—C14	119.75 (13)	C24—C25—H25B	109.6
C12—C13—H13	120.1	Br1—C25—H25B	109.6
C14—C13—H13	120.1	H25A—C25—H25B	108.1
			
C3—N2—C1—C4	126.19 (12)	C6—C7—C8—C9	−0.6 (2)
C23—N2—C1—C4	−52.97 (16)	C5—C4—C9—C8	−0.41 (19)
C3—N2—C1—C10	−103.07 (12)	C1—C4—C9—C8	−176.93 (12)
C23—N2—C1—C10	77.77 (15)	C7—C8—C9—C4	0.8 (2)
C3—N2—C1—C2	7.80 (13)	N2—C1—C10—C11	12.73 (16)
C23—N2—C1—C2	−171.36 (11)	C4—C1—C10—C11	141.71 (12)
C3—N1—C2—O1	−173.26 (12)	C2—C1—C10—C11	−95.46 (13)
C16—N1—C2—O1	−1.6 (2)	N2—C1—C10—C15	−174.51 (11)
C3—N1—C2—C1	8.82 (13)	C4—C1—C10—C15	−45.53 (15)
C16—N1—C2—C1	−179.55 (11)	C2—C1—C10—C15	77.30 (13)
N2—C1—C2—O1	172.39 (12)	C15—C10—C11—C12	0.05 (19)
C4—C1—C2—O1	53.90 (16)	C1—C10—C11—C12	172.84 (12)
C10—C1—C2—O1	−71.20 (15)	C10—C11—C12—C13	−0.7 (2)
N2—C1—C2—N1	−9.70 (12)	C11—C12—C13—C14	0.8 (2)
C4—C1—C2—N1	−128.19 (10)	C12—C13—C14—C15	−0.2 (2)
C10—C1—C2—N1	106.71 (11)	C13—C14—C15—C10	−0.5 (2)
C23—N2—C3—O2	−3.2 (2)	C11—C10—C15—C14	0.53 (19)
C1—N2—C3—O2	177.56 (14)	C1—C10—C15—C14	−172.36 (12)
C23—N2—C3—N1	176.11 (11)	C2—N1—C16—C17	−67.44 (15)
C1—N2—C3—N1	−3.09 (15)	C3—N1—C16—C17	103.23 (14)
C2—N1—C3—O2	175.42 (13)	N1—C16—C17—C22	131.78 (13)
C16—N1—C3—O2	3.7 (2)	N1—C16—C17—C18	−48.94 (16)
C2—N1—C3—N2	−3.96 (14)	C22—C17—C18—C19	−0.11 (19)
C16—N1—C3—N2	−175.66 (11)	C16—C17—C18—C19	−179.40 (12)
N2—C1—C4—C9	100.21 (13)	C17—C18—C19—C20	0.4 (2)
C10—C1—C4—C9	−28.97 (16)	C18—C19—C20—C21	−0.2 (2)
C2—C1—C4—C9	−148.14 (12)	C19—C20—C21—C22	−0.3 (2)
N2—C1—C4—C5	−76.28 (14)	C20—C21—C22—C17	0.6 (2)
C10—C1—C4—C5	154.53 (12)	C18—C17—C22—C21	−0.4 (2)
C2—C1—C4—C5	35.37 (15)	C16—C17—C22—C21	178.90 (12)
C9—C4—C5—C6	−0.2 (2)	C3—N2—C23—C24	−80.83 (15)
C1—C4—C5—C6	176.36 (12)	C1—N2—C23—C24	98.26 (14)
C4—C5—C6—C7	0.4 (2)	N2—C23—C24—C25	172.26 (11)
C5—C6—C7—C8	0.0 (2)	C23—C24—C25—Br1	178.82 (9)

Symmetry codes: (i) −*x*, *y*+1/2, −*z*+3/2; (ii) −*x*+1, *y*+1/2, −*z*+3/2; (iii) *x*, *y*−1, *z*; (iv) *x*−1, *y*, *z*; (v) −*x*, −*y*+1, −*z*+1.

### 3-Benzyl-1-(3-bromopropyl)-5,5-diphenylimidazolidine-2,4-dione Hydrogen-bond geometry (Å, º)

*Cg*4 is the centroid of the C17–C22 benzene ring.

**Table:**

*D*—H···*A*	*D*—H	H···*A*	*D*···*A*	*D*—H···*A*
C12—H12···O1^i^	0.95	2.49	3.2158 (17)	133
C16—H16*A*···*Cg*4^v^	0.99	2.66	3.5901 (14)	157
C25—H25*B*···*Cg*4^vi^	0.99	2.89	3.7621 (17)	148

Symmetry codes: (i) −*x*, *y*+1/2, −*z*+3/2; (v) −*x*, −*y*+1, −*z*+1; (vi) *x*, *y*+1, *z*.
